# Phenolic Composition and Antioxidant and Antiproliferative Activities of the Extracts of Twelve Common Bean (*Phaseolus vulgaris* L.) Endemic Ecotypes of Southern Italy before and after Cooking

**DOI:** 10.1155/2016/1398298

**Published:** 2016-12-25

**Authors:** Maria Neve Ombra, Antonio d'Acierno, Filomena Nazzaro, Riccardo Riccardi, Patrizia Spigno, Massimo Zaccardelli, Catello Pane, Mena Maione, Florinda Fratianni

**Affiliations:** ^1^Institute of Food Science, CNR-ISA, Via Roma 64, 83100, Avellino, Italy; ^2^Azienda “F. Marsocci”, Via Varignano 7, 80011 Acerra, Italy; ^3^CREA, Via dei Cavalleggeri 25, 84098 Pontecagnano, Italy

## Abstract

Beans are important dietary components with versatile health benefits. We analysed the extracts of twelve ecotypes of* Phaseolus vulgaris *in order to determine their phenolic profiles, antioxidant activity, and the* in vitro *antiproliferative activity. Ultra-performance liquid chromatography with diode array detector (UPLC-DAD) admitted us to detect and quantify some known polyphenols, such as gallic acid, chlorogenic acid, epicatechin, myricetin, formononetin, caffeic acid, and kaempferol. The antioxidant activity (AA) ranged from 1.568 ± 0.041 to 66.572 ± 3.197 mg necessary to inhibit the activity of the 2,2-diphenyl-1-picrylhydrazyl (DPPH) radical by 50% (EC_50_). The extracts, except those obtained from the nonpigmented samples, were capable of inhibiting the proliferation of the human epithelial colorectal adenocarcinoma (Caco-2) cells, human breast cancer cells MCF-7, and A549 NSCLC cell line. Cultivars differed in composition and concentration of polyphenols including anthocyanins; cooking affected the antioxidant activity only marginally. Qualitative and quantitative differences in phenolic composition between the groups of beans influenced the biological activities; on the other hand, we did not find significant differences on the biological activities within the same variety, before and after cooking.

## 1. Introduction

Among legumes, common beans (*Phaseolus vulgaris *L.) are widely consumed throughout the world. They play a significant role in human nutrition, being an important source of plant proteins, minerals, and certain vitamins and exhibit, for this reason, high nutritional value. In last years, bioactive effects, associated with the fibres, polyphenols, and other beans components related to human health have gained attention [1, 2]. Beans contain substantial amount of phenolic acids and flavonoids; some cultivars (red, black, and blue-violet coloured beans) show also anthocyanins, such as delphinidin and cyanidin, that overall attribute them a very strong antioxidant and antiradical activities [3]. Polyphenols are essentially present in the seed coats and in minor amount in cotyledons.

The beneficial effects of polyphenols on human health are expressed primarily through the reduction of the oxidative stress [4]. Some polyphenols are also able to exert antiapoptotic, antiaging, as well as anticarcinogenic activity, overall inhibiting the cell proliferation processes [5, 6].

Epidemiological studies have shown a strong link between the consumption of legumes and prevention of cancer risk, as well as the reduction of diabetes and cardiovascular risk through a normalization of the blood lipid and glucose profile [7]. Wedick et al. (2012) demonstrated that, among flavonoids, higher intakes of anthocyanins could be significantly associated with a lower risk of type 2 diabetes (risk: 0.85) [8].

Legumes are generally eaten without the elimination of the coat and in any case after cooking. Oroian and Escriche (2015) reported the antioxidant effect and total phenolic content of common beans, showing that pigmented beans had generally major antioxidant effect and higher amount of total polyphenols, with respect to the nonpigmented ones [9]. Due to their particular geographical characteristics, the territory of Southern Italy shows many different environments. During the past centuries, local farmers selected various bean seeds after their discovery in South America and diffusion in Europe. A so long activity of selection produced a small number of selected local cultivars, with unique characteristics. Studies regarding the effect of thermal processing on the health relevant functionality of beans are limited, especially with respect to the traditional cultivars present in those areas where the model of the Mediterranean diet is still strong. An improved information of the chemical composition and biological properties of local bean cultivars could help to define and valorise those with a greater nutraceutical potential to suggest to consumers.

Starting from such assumptions, aim of this study was to study the polyphenols (including total flavonoids and anthocyanins), the antioxidant activity, and the antiproliferative activity of the extracts obtained from some nonpigmented, red, speckled, and dark beans endemic ecotypes of Southern Italy. The study was carried out before and after the cooking domestic process.

## 2. Materials and Methods

**Figure 1:**
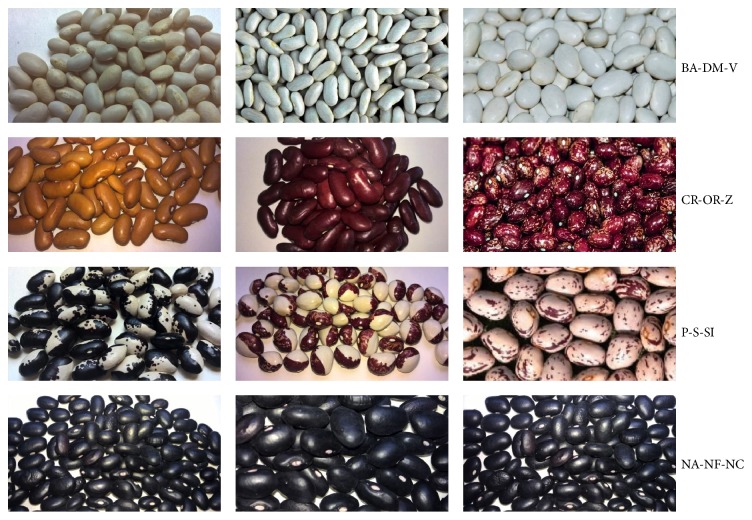
Photo of 12 varieties of beans (*Phaseolus vulgaris* L.) analyzed. Samples were classified into four groups: nonpigmented beans, red beans, speckled beans, and dark beans. Nonpigmented beans: Bianco Acerra (BA), Dente di morto (DM), and Volturara (V). Red beans: Cannellino Rosso (CR), ‘O Russ (OR), and Zampognaro (Z). Speckled beans: Pettilanculo (P), Sanghellatto (S), and Screziato impalato (Si). Dark beans: Nero di Acerra (NA), Nero di Frigento (NF), and Nero di Caposele (NC).

Twelve varieties of common bean (*Phaseolus vulgaris* L.) were collected in various rural communities from Campania and Basilicata regions of Southern Italy (Figure 1). Samples were divided into four bean groups based on the colour: nonpigmented beans, red beans, speckled beans, and dark beans; each group included three cultivars (Table 1). Dried beans were stored in the dark prior to extraction.

### 2.1. Sample Preparation

Samples (10 g) were rehydrated (1 : 5, w/v) for 24 hours; the water was discharged and 4 volumes of acetone were added. After 2 h of incubation at 4°C and centrifugation at 11,600 ×g (Biofuge, Beckman, Cassina de Pecchi, Italy), the supernatants were recovered and stored at 4°C. Pellets were treated again with one volume of acetone and incubated for 1 h at 4°C; the two supernatants were pooled, filtered, and, after a complete evaporation, stored at −20°C in the dark, until the analyses were performed [10]. The cooking of beans was performed with deionised water (1 : 5, w/v), in a home pressure cooker, for 20 min after the constant output of steam by the pressure safety valve. Cooked beans were immediately cooled, and the extracts were prepared as previously described.

### 2.2. Total Polyphenol Content

Total polyphenols (TP) of the extracts were determined using the Folin-Ciocalteu colorimetric method, as described by Singleton and Rossi Jr. [11]. Briefly, 50 *μ*L of extract was added to 50 *μ*L of Folin-Ciocalteu reagent in 800 *μ*L of distilled water. The reaction was neutralized with a sodium carbonate solution (20 g/100 mL). After incubation for 2 h at room temperature, the absorbance at *λ* = 760 nm was determined using a Cary UV/Vis spectrophotometer (Varian, Palo Alto, CA, USA). Quantification was based on a standard curve generated using gallic acid. The results were expressed as *μ*g of gallic acid equivalents (GAE)/g of DW samples ± standard deviation (SD).

### 2.3. Total Flavonoids

Total flavonoids (TF) were spectrophotometrically determined at 510 nm following the method of Zhishen et al. [12] with some modifications. The extract (50 *μ*L) was added to distilled water followed by 5% NaNO_2_ and after 5 min by AlCl_3_ 10%. After further 5 min, the reaction mixture was treated with 0.2 mL of 1 mM NaOH. Finally, the reaction mixture was diluted to 1 mL with deionised water and the absorbance was measured at *λ* = 510 nm (Varian, Palo Alto, CA, USA). TF amounts were expressed as *μ*g quercetin equivalents/gram of DW samples ± standard deviation (SD).

### 2.4. Anthocyanin Content

The amount of anthocyanins was determined by the differential pH method [13]. Absorbance was measured in a Cary UV/Vis spectrophotometer (Varian, USA), simultaneously at *λ* = 510 nm and *λ* = 700 nm in buffers of pH 1.0 and 4.5, using the formula *A* = (*A*510 − *A*700)_pH1.0_ − (*A*510 − *A*700)_pH4.5_. A molar absorption of 26,900 mol/cm was used for cyanidin-3-glucoside (molecular weight of 449.2 g/mol). Results were expressed as micrograms of cyanidin-3-glucoside equivalents/g of DW samples ± standard deviation (SD).

### 2.5. DPPH Radical-Scavenging Activity

The free radical-scavenging activity was determined using the stable radical 2,2-diphenyl-1-picrylhydrazyl (DPPH assay) [14]. The analysis was performed in microplates by adding 15 *μ*L of extract to 300 *μ*L of a methanolic DPPH solution (6 × 10^−5^ M). Next, the absorbance at *λ* = 517 nm was spectrophotometrically measured (Cary 50 MPR, Varian, USA). The absorbance of DPPH without antioxidant (control sample) was used for baseline measurements. The scavenging activity was expressed as the 50% effective concentration (EC50), which was defined as the sample amount (mg) necessary to inhibit DPPH radical activity by 50% during 60 min of incubation. These experiments were performed in triplicate, and the results were expressed as the mean values ± standard deviation.

### 2.6. Phenol Antioxidant Index (PAOXI)

As suggested by Vinson et al. [15] the phenol antioxidant index (PAOXI) can be used to estimate a combined measure of the quality and quantity of antioxidants present in a sample. In this study, it was calculated by dividing the DPPH radical-scavenging activity of the sample (*μ*mol of DPPH inhibited/mg) for the total phenol concentration (mg/g) [16].

### 2.7. Chromatographic Analysis

An ACQUITY Ultra-Performance LC system (Waters) linked to a PDA 2996 photodiode array detector (Waters) was used for ultra-performance liquid chromatography analyses. The Empower software controlled the instruments and acquired and processed the data. The extracts and standards (previously dissolved in methanol) were filtered (0.45 *μ*m; Waters) before analysis. The analyses were carried at 30°C using a reversed phase column (BEH C_18_, 1.7 *μ*m, 2.1 100 mm; Waters) following the method of Fratianni et al. [17]. The mobile phase consisted of solvent A (7.5 mM acetic acid) and solvent B (acetonitrile) at a flow rate of 250 *μ*L min^−1^. Gradient elution was employed, starting with 5% B for 0.8 min; then 5–20% B over 5.2 min; isocratic 20% B for 0.5 min; 20–30% B for 1 min; isocratic 30% B for 0.2 min; 30–50% B over 2.3 min; 50–100% B over 1 min; isocratic 100% B for 1 min; and 100–5% B over 0.5 min. At the end of this process, the system equilibrated the column under the initial conditions for 2.5 min.

### 2.8. Determination of* In Vitro* Cancer Cell Viability

#### 2.8.1. Cell Culture and Extracts

MCF-7, A549, and Caco-2 cell lines were obtained from the American Type Culture Collection (ATCC, Rockville, MD). Cells were grown in Dulbecco's modified eagle's medium (DMEM) supplemented with 10% of heat-inactivated foetal bovine serum (FBS), penicillin (0.010 U L^−1^), streptomycin (10 mg L^−1^), and sodium pyruvate 0.0002 M. Cultures were incubated in the presence of 5% CO_2_ at 37°C and 100% relative humidified atmosphere. Several concentrations of extract were dissolved in DMEM medium. The culture media were evaluated in terms of pH and precipitation of components, in order to avoid possible cytotoxic effects due to changes in these factors on the cellular microenvironment. Only the concentrations that did not change the culture media conditions were selected for further assays.

#### 2.8.2. Crystal Violet Assay

The cells were plated (10 × 10^3^/well) in 48-well plates in complete medium. After 24 h, the medium was removed and replaced on the first day with the same medium (control) or supplemented with various extracts doses. Cells were observed with an inverted microscope every 24 h, to identify morphological changes, toxicity, or cell death. The percentage of cell proliferation was estimated on the fourth day by a colorimetric assay reported by Kueng et al. [18] modified as follows: cells were fixed for 20 min at room temperature with 1% paraformaldehyde (PFA), stained with 0.1% crystal violet in 20% methanol for 20 min, washed with PBS, solubilized with 10% acetic acid, and read at 595 nm in a microplate reader (Cary 50 MPR, Varian, USA). The experimental conditions were tested in triplicate, and separate experiments were performed on at least three separate occasions.

#### 2.8.3. 3-(4,5-Dimethylthiazol-2-yl)-2,5-diphenyltetrazolium Bromide (MTT) Reduction Assay

Cells were seeded in 96-well microplates at a density of 5 × 10^3^ cells/well and grown for 24 h at 37°C in 5%  CO_2_ prior to the addition of test samples. The cells were treated with different sample concentrations dissolved in Dulbecco's phosphate buffered saline (PBS). After 48 h of incubation, cell viability was determined using a colorimetric MTT assay. Cell survival (%) was measured as the reduction of MTT in formazan at 550 nm. Triton® X-100 (10 *μ*L of 10% solution) was used as a positive control. Untreated cells (vehicle alone) were chosen as the negative control. The controls and samples were assayed in triplicate for each concentration and replicated three times. The absorbance values were converted into percentages of cell viability using the following formula: Cell viability = Abs sample/Abs control × 100.

### 2.9. Statistical Analysis

Data were expressed as mean ± standard deviation of triplicate measurements. Analysis of variance (ANOVA) was used to compare results and significance was accepted at *p* < 0.05. The PC software “Excel Statistics” was used for the calculations. Principal Component Analysis (PCA) was used to relate values of biological activities to phenolic composition, using the free software environment for statistical computing and graphics R (https://www.r-project.org/). For cell assays, the EC50 values were calculated using ED50plus v1.0 online software.

## 3. Results and Discussion

### 3.1. Polyphenol Content

The content of total polyphenols (TP) present in the extracts of beans is shown in Table 2. Results, expressed as *μ*g GAE/gr (dw of seeds), showed significant differences (*p* ≤ 0.01) among samples; on the other hand, such differences were not significant (*p* > 0.05) within the same group of beans. Maximal quantitative differences in TP were obtained between the extracts of white beans (ranging from 135.04 to 275.4) and speckled beans (ranging from 1061.9 to 1249.7), with an even ratio of about 1 : 10. In the extracts of red and black beans, values of TP were intermediate, in agreement to the results reported by Heimler et al. [19], which studied other Italian varieties of* Phaseolus vulgaris* L. An exception was represented by the extract of* Cannellino Rosso* (CR) (TP = 229.70) which, although belonging to the red group, exhibited an amount of polyphenols comparable to that found in nonpigmented beans. The colour of this variety was more clear compared to the other red beans (Figure 1).

The extracts obtained from the cooked beans showed a lower amount of TP with respect to the corresponding raw samples, except those obtained from the nonpigmented beans, for which the amount of polyphenols remained similar after cooking. For example, taking into consideration the cultivar* Bianco di Acerra*, the amount of TP remained virtually the same, before and after the thermal treatment (275.442 and 241.782, resp.).

### 3.2. Determination of Flavonoids

Usually, flavonoids and other phenolic compounds are typically stored in the seed coat due to their antipathogen and antifeeding activities; furthermore, such localization assures the best protection of the seed from external attacks (pathogens, insects, etc.) [20]. Many of the flavonoids that give rise to the coat colour of beans may also provide positive health benefits as antioxidants. This class of polyphenols was present in all samples analysed, being particularly abundant in the coloured beans, with a very similar trend to that of the polyphenols, as shown in Table 2. The extracts of white beans showed a low content of flavonoids, not more than 58.73 *μ*g/QE. The highest quantities of flavonoids were found in the extracts of speckled beans as well as in that of the red cultivar ‘*O Russu* (OR). The extracts obtained from the three speckled beans* Pettilanculo *(P),* Sanghellatto* (S), and* Screziato impalato* (SI) showed 925.654 *μ*g/QE, 910.551 *μ*g/QE, and 703.336 *μ*g/QE, respectively; the extract of cultivar* Nero di Acerra* (NA) was the richest in terms of content in flavonoids among dark beans analysed (542.337 *μ*g/QE).

### 3.3. Determination of Total Anthocyanins

As expected, a higher content of anthocyanins was observed in the extracts of dark beans, while lower amounts were found in those of speckled and red samples, except* Zampognaro *(Z) bean (10.706 *μ*g C3GE/gram) (Table 2). The extract of the dark bean* Nero di Caposele* (NC) showed the highest value (63.278 *μ*g C3GE/gram) of anthocyanins; moreover, this cultivar proved to be also the richest with regard to total polyphenols and flavonoids among the dark cultivars analysed. It should be emphasized that the anthocyanins in the beans are exclusively present in the peel [21]. Therefore it is sufficient that the seeds are smaller to obtain a higher anthocyanin value, as we observed in our test as regard as cultivar NC. Cooking lowered the content of anthocyanins present in the samples; however, some cultivars (Z among speckled and NC among dark cultivars) maintained a content of anthocyanin of 6.11 *μ*g C3GE/gram and 10.508 *μ*g C3GE/gram, respectively.

### 3.4. Antioxidant Activity

The antioxidant effects in bean extracts were investigated by DPPH test. Overall, the samples exhibited relevant antioxidant qualities and few mg of the extracts were sufficient to inhibit at 50% of the activity of 1 mL of the free radical DPPH (Table 2). Speckled beans showed the highest antioxidant activity (EC_50_ not exceeding 1.813 mg/mL), followed by red beans (except for CR) and black beans (EC_50_ not superior than 5.2 mg/mL). As expected, the extracts of nonpigmented varieties exhibited the lowest antioxidant activity (with EC_50_ values ranging between 23.62 mg/mL and 55.21 mg/mL). Cooked beans, in spite of a loss of total polyphenols, disclosed intriguing results. To estimate the combined measure of the quality and quantity of antioxidants present in the sample, we adopted the so-called phenol antioxidant index (PAOXI) as suggested by Vinson et al. [15]. In our study, such index was calculated by dividing the DPPH radical-scavenging activity of the sample (expressed in this case as *μ*mol of DPPH inhibited/gram of product) with respect to the total polyphenol concentration (mg/g). This was possible by converting the values obtained in the antioxidant activity in mg, to *μ*M DPPH/mg sample. The highest PAOXI value (36.25) was exhibited by the extract of the speckled bean* Sanghellatto* (S); therefore, the extract of the dark cultivar* Nero di Caposele* exhibited the lowest PAOXI value (10.79) among coloured beans (Table 3). This simple calculation could help in clarifying about the relationship between polyphenols and antioxidant activity that is not always so direct.

Although it was possible to establish a clear correlation between the levels of polyphenols and the free radical-scavenging properties of the extracts, we observed some diversity, which could be ascribable to the differences between their components constituting total polyphenols. It is known that there are a variable number of combinations of the compounds for each sample and that the antioxidant potential of these individual components is variable. From the analysis of total polyphenols, total flavonoids, and antioxidant activity, we observed that the extracts of the dark bean* Nero di Caposele* (NC) showed the highest amount of total polyphenols; however, speckled* Screziato impalato* bean (SI), although containing slightly less polyphenols, exhibited higher flavonoid content, and its PAOXI index was significantly higher (10.79 versus 29.0). This reinforced the idea that the antioxidant potential could be strongly linked to the content of flavonoids. Cooking affected the antioxidant activity; for some varieties (e.g., NA and NF) the PAOXI values were similar to those obtained before cooking; indeed in some cases (NC) a higher PAOXI index was observed, suggesting that the thermal processing reinforced their antioxidant efficacy.

### 3.5. Identification of Polyphenols

**Figure 2:**
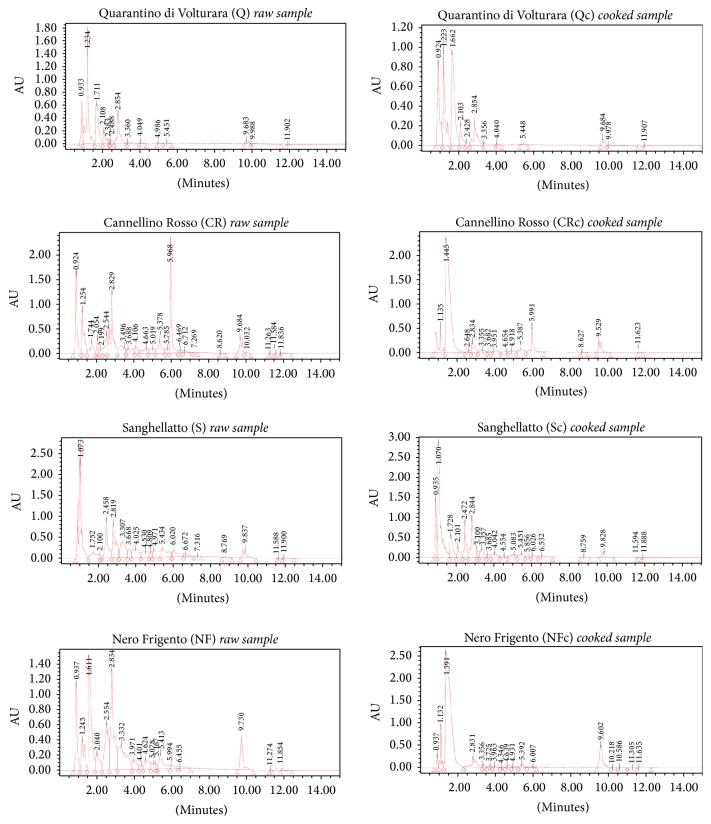
UPLC profiles of the polyphenols present into four bean groups: nonpigmented beans Quarantino di Volturara raw and cooked (Q-Qc); red beans Cannellino Rosso raw and cooked (CR-CRc); speckled beans Sanghellatto raw and cooked (S-Sc); and dark beans Nero Frigento raw and cooked (NF- NFc).

The UPLC analysis provided an overview of the main phenolic compounds in every sample. In Figure 2 we reported the chromatograms obtained from the extracts of four cultivars of beans, each of which representing a group (white, speckled, red, and dark). For each one, we reported the profiles obtained before and after the process of cooking.  Table 4 shows, organised as tabular form, the amount of each polyphenol recognised from the apparatus, on the basis of known standard. The data are separated according to the groups.

The profiles of the extracts of nonpigmented beans were very simple; in fact, we found only few peaks, present in the initial part of the chromatogram. The extract of the variety* Dente di Morto* DM showed even a minor number of peaks with respect to the other two nonpigmented beans BA and Q analysed (chromatogram not shown). Despite a few peaks viewable, the sensitivity of the equipment still allowed us to detect and identify several known polyphenols, even if they were present in small quantities in the nonpigmented beans (Table 4(a)). Polyphenol profiles of the extracts of speckled, red, and dark beans were richer and more complete. Some polyphenols were present only in the extracts giving rise from the pigmented beans. For example, myricetin was identified only in the extracts of the pigmented beans: it was present in the extract of raw bean NC (1.986 *μ*g/gram, Table 4(d)) and in the extracts of Z and Si after cooking (3.35 *μ*g/gram and 0.218 *μ*g/gram, Tables 4(c) and 4(b), resp.). Overall, our results were in agreement with dosage of polyphenols previously reported for beans, especially as regards flavonoids [22, 23]. We analysed more extensively those flavonoids already reported in literature for their antiproliferative activity [24], and we found appreciable amount of some flavones and/or flavonoids, such as formononetin, genistein, quercetin 3,4-diglucoside, spiraeside, and hyperoside, in the extracts of red samples (Table 4(b)). The extract of* Cannellino Rosso*, while containing a modest amount of total polyphenol with respect to the extracts of the other two red cultivars, exhibited a polyphenol profile absolutely well correlated with respect to the extracts of the other two red varieties. It should also be highlighted that the decrease of total polyphenols after the cooking of beans did not affect the amount of flavonoids, such as genistein and formononetin. Formononetin is a typical bioactive isoflavone of red clover plant with potent pharmacological activities, including antioxidant, antiviral, antitumor, and cardio protective effects [25]. In addition, this isoflavone is capable of inhibiting the proliferation, inducing apoptosis in human cancer cells, such as breast cancer, prostate tumour, and osteosarcoma [26, 27]. A study by HPLC performed on 62 wild and weedy Mexican bean ecotypes identified kaempferol as one of the main flavonoids, assuming that its quantitative variation could be more related to the genotype than to the seed coat colour [28]. In our experiments, flavonols, such as quercetin and kaempferol, were found both in raw and in processed beans. Likewise, we found kaempferol in the extracts of red cultivar* Zampognaro* (Z) and in those of the two speckled P and Si cultivars (Tables 4(b) and 4(c)). Kaempferol is one of the phenolic compounds more studied for its antimutagenic and anticarcinogenic activity both* in vitro *and* in vivo *[29]; its concentration in* P. vulgaris* is very variable, ranging from traces < 0.2 mg/kg (in Tuscany landraces) [30] to 209.4 mg/kg (in Mexican cultivated varieties) [3]. Our data were more in agreement with the Tuscany varieties [31]. It is to underline the presence of these compounds in cooked beans, so that they can really carry out their nutraceutical properties, above all because the flavonols are not much affected by heat treatment [32].

The extracts of the three speckled beans, in addition to flavonoids, exhibited many caffeic acid derivatives and an appreciable amount of catechin derivatives, including epigallocatechin (Table 4(c)).

Flavonoids showed a great variability; in fact, besides the formononetin and derivatives of quercetin, they contained also genistein and daidzein, at low amounts. Antiproliferative activity of flavonoids is well known and analysed in many studies [33, 34]. After cooking, the extracts of both* Pettilanculo* (P) and* Sanghellatto* (S) showed very high values of formononetin; it is also to underline the presence of spiraeoside in the extract of* Sanghellatto* after cooking (Sc, Table 4(c)).

The extracts of dark beans gave a well-varied profile, with appreciable values for formononetin and the presence of quercetin glucosides 3-4 (Table 4(d)).

### 3.6. Anti-Proliferative Activity

The antiproliferative activity of the extracts was evaluated on MCF-7 breast cancer cell line, A549 NSCLC cell line, and Caco-2 colon carcinoma cells using a MTT colorimetric test. After 48 h of treatment, growth of cells was significantly inhibited in a dose-dependent manner, and, for each extract, an EC50 value was calculated from dose-response curves of the cell lines. In Table 3, EC50 values on three cell lines are reported, indicating the concentration of the extracts (expressed as *μ*g equivalent to gallic acid, GAE, as a standard, of total polyphenols present in the extract/mL) able to reduce cell viability by 50%. The extracts of the nonpigmented varieties (BA, DM, and V) did not cause a significant reduction of the cell viability, for each cell line, even at the highest concentration used; the data are not shown and these varieties were not included in Table 5. The EC50 values varied strongly among the three cell lines and the different extracts tested, indicating a different cellular sensitivity to the extracts, as illustrated from the EC50 values. However, the ANOVA analysis on the mean EC50 values obtained for the three cell lines did not return significant *p* value (*p* > 0.05). The highest EC50 values were measured in A549 cancer cells, using the extract of variety* Pettilanculo*, while the lowest was found in CaCo-2 cells, with CR extracts. Generally, the concentrations need to inhibit the proliferation of Caco-2 cells which were 1.06–3.64 folds lower than those of A549 and MCF-7 cells without exception. Overall, the extracts of P and Pc showed only moderate inhibitory effect against the three cancer cell lines, while those of CR and NFc were the most active. In addition, the extracts of cooked beans were less efficient than the correspondent crude extracts, except for Pc, NFc, and NCc, which showed lower EC50 values than the analogous crude extracts. Likely, in these cases, the thermal processing released compounds with inhibitory capacities. Overall, for the same cell line, the values of the inhibition obtained with raw samples were not significantly different with respect to the cooked counterparts.

### 3.7. Principal Component Analysis

**Figure 3:**
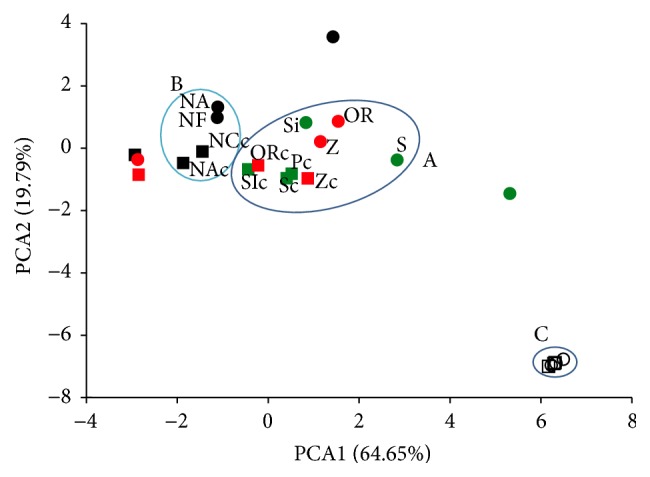
Principal component analysis (PCA): distribution of 12 raw and 12 cooked bean varieties along principal components 1 (PC1) and 2 (PC2). using 7 variables. White circles indicate nonpigmented raw varieties and white squares, nonpigmented cooked varieties. Red circles represent red raw cultivars, red squares for red cooked beans, green circles for speckled raw beans, green squares for speckled cooked beans, black circles for dark raw beans, and black squares for dark cooked beans.

We applied principal component analysis (PCA) to characterize the twelve varieties of beans according to their biological activity. The method was successfully used in the treatment of data in the study of apples [35] and black beans [36]. In our study, seven variables were measured in 24 samples (12 raw and 12 cooked beans), and data were analysed by PCA (Figure 3). The cumulative percentage of the total variance explained by the first two components was 84.4%. A bidimensional plot was designed (Figure 3). The distribution of the varieties along PC1 and PC2 showed that samples could be divided into three main groups: group A, which included red varieties and speckled beans positioned near to the centre of the bidimensional plot; group B, which comprised black varieties, and group C, corresponding to exclusively nonpigmented varieties. Group A included beans with the highest concentrations of phenolic compounds and the best antioxidant activity. These varieties differed from the others due to their higher content of total polyphenols, in particular, flavonoids, which were able to influence more effectively both the antioxidant activity and the capability to inhibit the growth of the cancer cells. In fact, we found a positive correlation (*r* > 0.736) between the total concentration of polyphenols and flavonoids and the antioxidant and antiproliferative activities. The highest content of anthocyanins was detected in the extracts of black beans (Table 2). Herein, it is important to underline that such variable contributed to distinguish this group B from the others (Figure 3). Group C included the extracts of nonpigmented beans, which exhibited lower phenolic contents and antioxidant activities correlated to nonsignificant antiproliferative effects.

Our analysis suggested that flavonoids and anthocyanins were accountable for the antioxidant and antiproliferative effects of these extracts. The most active varieties were those belonging to the speckled group. The red varieties were only slightly less active and overlapping with speckled group in PCA. The extracts of dark beans, despite a higher content of anthocyanins, exhibited lower AA values than the speckled, suggesting that the flavonoids were mostly responsible for such biological activity.

## 4. Conclusions

Common beans are a key food of the Mediterranean diet, representing an important source of proteins, fibres, some minerals, vitamins, and bioactive compounds. We evaluated the antioxidant and antiproliferative effects of polyphenolic extracts obtained from old twelve varieties of endemic bean (*Phaseolus vulgaris* L.), of some rural countries of Southern Italy. Our findings confirm that the speckled, red, and black coloured varieties of beans are more active than the nonpigmented ones. Interestingly, cooking of beans, despite causing a certain loss of bioactive molecules, did not negatively affect in marked way their biological properties. This undoubtedly may represent a result of considerable importance, in order to safeguard and promote the old varieties of beans for their important nutraceutical potential, enhancing their market opportunities in the production of functional food and nutraceuticals.

## Figures and Tables

**Table 1:** Sample classification.

Raw sample	Cooked sample
(1a) Not pigmented beans: Bianco Acerra (BA)	(1a′) Not pigmented beans: Bianco Acerra (BAc)
(1b) Not pigmented beans: Dente de Morto (DM)	(1b′) Not pigmented beans: Dente de Morto (DMc)
(1c) Not pigmented beans: Volturara (V)	(1c′) Not pigmented beans: Volturara (Vc)

(2a) Red beans: Cannellino Rosso (CR)	(2a′) Red beans: Cannellino Rosso (CRc)
(2b) Red beans: O Russ (OR)	(2b′) Red beans: O Russ (ORc)
(2c) Red beans: Zampognaro (Z)	(2c′) Red beans: Zampognaro (Zc)

(3a) Speckled beans: Pettilanculo (P)	(3a′) Speckled beans: Pettilanculo (Pc)
(3b) Speckled beans: Sanghellatto (S)	(3b′) Speckled beans: Sanghellatto(Sc)
(3c) Speckled beans: Screziato impalato (Si)	(3c′) Speckled beans: Screziato impalato (Sic)

(4a) Dark beans: Nero Acerra (NA)	(4a′) Dark beans: Nero Acerra (NAc)
(4b) Dark beans: Nero Frigento (NF)	(4b′) Dark beans: Nero Frigento (NFc)
(4c) Dark beans: Nero Caposele (NC)	(4c′) Dark beans: Nero Caposele (NCc)

Cooked samples are indicated with “c.”

**Table 2:** Total polyphenols (PT), antioxidant activity, flavonoids (FT), and anthocyanin contents in the bean extracts. The concentration of total polyphenols was expressed as *µ*g equivalents of gallic acid/g of DW sample; antioxidant activity was expressed as EC50 (mg/mL DPPH); flavonoids were expressed as *µ*g equivalents of quercetin/g of DW sample; the content of anthocyanin content was expressed as *µ*g equivalents of cyanidin-3-glucoside/g of DW sample. Data are the mean values of three independent experiments (±standard deviation).

	Polyphenol content (*µ*g GAE/gr (DW))	Antioxidant activity (mg × mL di DPPH)	Flavonoids content (*µ*g (QE)/gr (DW))	Anthocyanin content (*µ*g (C3GE)/gr (DW))
(BA)	275.442 ± 58.621	23.619 ± 0.532	54.733 ± 3.316	0
(DM)	135.037 ± 6.316	55.217 ± 5.178	58.731 ± 2.471	0
(V)	165.524 ± 12.77	28.826 ± 2.329	51.511 ± 1.422	0
(BA-c)	241.782 ± 54.213	61.605 ± 8.087	50.992 ± 4.804	0
(DM-c)	211.907 ± 37.017	50.133 ± 3.786	34.323 ± 4.798	0
(V-c)	126.29 ± 20.212	66.572 ± 3.197	49.483 ± 5.883	0

(CR)	229.698 ± 3.909	10.619 ± 0.211	100.151 ± 9.367	3.015 ± 0.691
(OR)	1205.446 ± 23.884	1.722 ± 0.013	878.749 ± 58.878	8.864 ± 0.773
(Z)	997.774 ± 90.482	1.927 ± 0.036	562.378 ± 48.128	10.706 ± 0.561
(CR-c)	182.275 ± 23.009	27.291 ± 3.351	116.189 ± 2.822	1.148 ± 0.068
(OR-c)	621.338 ± 27.434	6.022 ± 0.145	360.417 ± 4.864	5.323 ± 0.248
(Z-c)	687.874 ± 25.338	4.849 ± 0.045	202.396 ± 11.1	6.113 ± 0.763

(P)	1249.71 ± 29.941	1.583 ± 0.047	925.654 ± 90.209	6.314 ± 1.285
(S)	1061.935 ± 13.603	1.568 ± 0.041	910.551 ± 137.472	3.629 ± 0.193
(Si)	1150.474 ± 15.993	1.813 ± 0.056	703.336 ± 121.5	7.255 ± 1.794
(P-c)	607.606 ± 8.486	5.052 ± 0.171	332.618 ± 6.633	2.388 ± 0.796
(S-c)	565.326 ± 58.081	3.82 ± 0.003	192.021 ± 21.969	1.338 ± 0.372
(Si-c)	622.14 ± 74.146	6.253 ± 0.116	245.306 ± 22.634	3.963 ± 0.456

(NA)	604.23 ± 53.995	5.161 ± 0.352	244.698 ± 14.524	29.702 ± 3.327
(NF)	601.269 ± 52.156	4.346 ± 0.088	293.928 ± 9.119	18.909 ± 2.457
(NC)	1291.62 ± 40.16	4.347 ± 0.609	542.337 ± 16.454	63.278 ± 1.547
(NA-c)	359.844 ± 28.37	7.996 ± 0.042	112.927 ± 2.86	5.63 ± 0.799
(NF-c)	240.553 ± 23.097	14.528 ± 0.803	99.694 ± 4.163	6.939 ± 0.608
(NC-c)	436.626 ± 50.194	6.78 ± 0.041	193.103 ± 9.974	10.508 ± 1.206

**Table 3:** The phenol antioxidant index (PAOXI) (*µ*mol of DPPH inhibited/mg) by the total phenol concentration (mg/g). Data are the means values of three independent experiments (± standard deviation).

PAOXI		
	Raw sample	Cooked sample
(1a) (BA)	9.486	4.016
(1b) (DM)	8.081	5.687
(1c) (V)	12.987	7.215

(2a) (CR)	24.61	12.076
(2b) (OR)	28.71	16.05
(2c) (Z)	31.344	18.045

(3a) (P)	30.187	19.574
(3b) (S)	36.25	27.8
(3c) (Si)	29.002	15.434

(4a) (NA)	19.252	20.918
(4b) (NF)	23.003	17.206
(4c) (NC)	10.709	20.297

**(a):** 

	(1a) (BA)	(1a) (BAc)	(1b) (DM)	(1b) (DMc)	(1c) (V)	(1c) (Vc)
Gallic acid	64.563	64.827	53.168	116.329	49.236	58.734
Caftaric acid	14.247	7.002			7.907	4.021
Chlorogenic acid	104.362	96.367	64.453	83.111	65.225	29.6
Epigallocatechin		4.087				
Cichoric acid	11.022					
Catechin	10.002				9.411	6.068
Caffeic acid				0.539		
Epicatechin	2.603	2.093		0.824	1.807	1.16
Sinigrin				1.779	1.939	
Coumaric acid	1.777	1.587				
Hyperoside	8.83	9.08	4.532	3.453	2.536	20.823
Spiraeoside	3.643					
Formononetin	26.208	66.753	6.989	1.253	2.682	4.172

**(b):** 

	(2a) (CR)	(2a) (CRc)	(2b) (OR)	(2b) (ORc)	(2c) (Z)	(2c) (Zc)
Gallic acid	67.565	60.985	259.764	56.526	618.536	555.525
Caftaric acid	15.55	6.848		6.559	49.795	33.45
Chlorogenic acid	76.245	35.456	308.9	67.963	132.459	82.863
Epigallocatechin						35.165
Cicoric acid		21.73	160.473	35.504	117.773	
Catechin	22.772					
Vanillic acid	11.855	5.87	38.845	6.959		
Epicatechin	3.27	4.4	35.573	5.926	13.191	3.373
Quercetin 3,4 diglucoside				1.941	17.595	
1-3 dicaffeoylquinic acid				17.311		
Sinigrin	5.312				16.668	1.193
Siringaldeid	15.045					
Hyperoside		24.541	205.9	27.281	28.773	4.323
Taxifolin	2.48		43.945			
Indole3 carboxilic a.				0.526		0.27
Spiraeosid			295.418	10.019		
Miricetin						0.218
Genistein	2.09	3.651				
Kampherol					6.723	6.045
Formononentin	38.648	32.289	25.736	24.985	4.986	4.063

**(c):** 

	(3a) (P)	(3a) (Pc)	(3b) (S)	(3b) (Sc)	(3c) (Si)	(3c) (Sic)
Gallic acid	301.135	416.078	521.645	292.937		428.594
Caftaric acid	203.988	76.12	145.791	49.122	87.6	39.629
Chlorogenic acid	141.223	86.658	325.264	103.334	237.473	73.644
Epigallocatechin	27.028			12.285		
Cicoric acid	137.253	67.065	156.018	79.144	288.118	47.206
Catechin			59.173			
Vanillic acid	62.34		38.509		63.736	
Caffeic acid						2.488
Epicatechin	21.388	9.815	34.427	8.717	56	
Quercetin 3,4 diglucoside			34.809		57.5	4.91
1-3 dicaffeoylquinic acid	82.608					
Sinigrin	47.455	12.978		12.793	154.191	
Hyperoside	60.343	21.27	32.073	22.749	146.036	6.567
Rosmarinic acid	36.758				56.964	2.502
Taxifolin		14.038	9.127			
Spiraeosid	113.72			39.507		
Miricetin					3.355	
Dadzein	5.67				8.218	
Luteolin				3.095		
Quercetin	8.595					
Genistein		3.732				
Naringenin				0.622		
Kampherol	1.98				5.727	5.219
Formononentin	54.108	53.785	21.373	12.951	4.527	3.642

**(d):** 

	(4c) (NC)	(4c) (NCc)	(4b) (NF)	(4b) (NFc)	(4a) (NA)	(4a) (NAc)
Gallic acid	38.359	176.217	74.851	79.488	186.945	83.053
Caftaric acid	90.605	38.329	74.017	26.932		41.02
Chlorogenic acid	334.545	122.52	178.263	94.236	752.809	166.8
Cicoric acid			153.017			54.333
Catechin				7.38		
Vanillic acid					29.8	
Siringic acid		0.951		1.296		1.887
Epicatechin	14.986	0.871	10.9	1.488	34.482	
Quercetin 3,4 diglucoside	45.691	5.526	11.229			
Sinigrin				1.936		
Coumaric acid	6.177		4.377		14.791	3.047
Ellagic acid			18.083			
Siringaldeid	30.8	4.311			113.891	17.72
Hyperoside			34.506	6.68		
Taxifolin	13.659				21.782	
Indole 3 carboxiladeid					5.245	1.18
Miricetin	1.986					
Genistein						9.467
Formononentin	35.941	28.137	39.72	5.096	163.336	65.62
Biochanin-A						4.12

**Table 5:** The antiproliferative activity of the extracts on MCF-7, A549, and Caco-2 cells. EC50 values indicate the concentration (expressed as *μ*g equivalent of gallic acid of total polyphenols/mL) able to reduce cell viability by 50% (±SD).

Sample	MCF-7 cell line EC50 *μ*g GAE mL^−1^ (±SD)	CaCo-2 cell line EC50 *μ*g GAE mL^−1^ (±SD)	A549 cell line EC50 *μ*g GAE mL^−1^ (±SD)
CR	29.40 (1.13)	28.52 (4.9)	31.32 (4.2)
CRc	44.17 (2.7)	37.49 (6.37)	68.79 (0.61)
OR	128.17 (1.17)	58.05 (4.16)	130.83 (2.92)
Orc	135.81 (0.54)	77.62 (2.23)	121.09 (2.84)
Z	185.44 (2.04)	49.85 (6.01)	177.96 (0.61)
Zc	196.70 (1.7)	66.29 (3.11)	273.08 (4.69)
P	333.37 (2.3)	186.18 (14.11)	377.40 (8.34)
Pc	115.33 (1.6)	72.23 (8.72)	252.81 (2.28)
S	165.57 (0.52)	100.17 (2.36)	293.62 (6.75)
Sc	181.36 (0.47)	55.65 (2.9)	213.27 (7.16)
Si	102.60 (0.84)	44.46 (3.73)	122.43 (1.5)
Sic	158.61 (2.7)	76.50 (2.39)	88.01 (1.15)
NA	67.64 (1.06)	53.75 (1.47)	49.40 (0.55)
NAc	82.57 (0.11)	55.46 (2.02)	55.80 (1.26)
NF	63.75 (1.3)	35.79 (4.39)	54.0 (5.51)
NFc	33.6 (1.27)	29.4 (3.96)	31.92 (3.08)
NC	119.02 (1.69)	73.53 (2.36)	118.35 (1.63)
NCc	107.16 (1.03)	52.97 (1.87)	49.486 (2.56)
